# First steps into the pupillometry multiverse of developmental science

**DOI:** 10.3758/s13428-023-02172-8

**Published:** 2023-07-13

**Authors:** Giulia Calignano, Paolo Girardi, Gianmarco Altoè

**Affiliations:** 1 Department of Developmental and Social Psychology, University of Padua, Padua, Italy; 2 Department of Environmental Sciences Informatics and Statistics, Ca’ Foscari University, Venice, Italy

**Keywords:** Multiverse, Pupillary response, Preprocessing, Infancy

## Abstract

Pupillometry has been widely implemented to investigate cognitive functioning since infancy. Like most psychophysiological and behavioral measures, it implies hierarchical levels of arbitrariness in preprocessing before statistical data analysis. By means of an illustrative example, we checked the robustness of the results of a familiarization procedure that compared the impact of audiovisual and visual stimuli in 12-month-olds. We adopted a multiverse approach to pupillometry data analysis to explore the role of (1) the preprocessing phase, that is, handling of extreme values, selection of the areas of interest, management of blinks, baseline correction, participant inclusion/exclusion and (2) the modeling structure, that is, the incorporation of smoothers, fixed and random effects structure, in guiding the parameter estimation. The multiverse of analyses shows *how* the preprocessing steps influenced the regression results, and *when* visual stimuli plausibly predicted an increase of resource allocation compared with audiovisual stimuli. Importantly, smoothing time in statistical models increased the plausibility of the results compared to those nested models that do not weigh the impact of time. Finally, we share theoretical and methodological tools to move the first steps into (rather than being afraid of) the inherent uncertainty of infant pupillometry.

## Introduction

Over the last decade, infant research have blended traditional measures (e.g., saccade latency and number of fixations) of cognitive functioning with pupillometry, a reliable and fine-graded index of attentional and perceptual mechanisms from infancy (for a review see Hepach & Westerman, 2016) to adulthood (Laeng et al., 2012; Kucewicz et al., 2018). Importantly, the pupil transient and event-locked phasic response reflect active engagement on events (Laeng et al., 2012) and is a promising supplement of more established measures such as looking times (Jackson & Sirois, 2022; for a review on the topic see Hepach & Westermann, 2016). As any eye-tracking measure, pupillometry studies generate rich time series datasets, with thousands of values per participant (according to the refreshing rate usually ranging from 20 to 1000 Hz; for a debate see Mathot & Vilotijević, 2022), in which diameter changes over time can be thought as a nonlinear signal varying across time. Such variation in pupil size involves both the autonomic and somatic nervous systems associated with activation of the locus coeruleus. Pupil dilation is considered an impartial and involuntary marker of central nervous system activity, as shown by brain activity recorded on the scalp with EEG (for a review, see Hepach & Westermann, 2016; Patwari et al., 2012), and it reflects cognitive functions such as attention, arousal, and cognitive load (Beatty, 1982; Karatekin et al., 2004; Porter et al., 2007). 

By capitalizing on an illustrative research question, we took advantage of the multiverse approach to data analysis to check the robustness of results in cognitive pupillometry applied to infancy research. The main idea was to use pupillometry as a marker of attention deployment toward novel visual and audiovisual information (Hollich et al., 2007; Cheng et al., 2019), in 12-month-old infants. It was expected that just a few exposures to an audiovisual (vs. visual) stimulus should have increased the attentive response indexed by increased pupil dilation depending on the familiarization type. Specifically, a higher pupil phasic response should indicate an increase in resource allocation and information encoding (Cheng et al., 2019).

## The multiverse has always been there: The issue of building datasets

On the one hand, while dealing with behavioural and especially psychophysiological data, we face a wide range of challenges in selecting a rationale that minimizes data manipulation by *letting data talk*. On the other hand, with varying degrees of awareness, we are also obliged to make decisions about a dataset structure, in order to organize information and make it usable for data analysis. Preprocessing steps are arbitrary choices that can dramatically drive the results (Steegen et al., 2016). Furthermore, when such sophisticated choices are not shared with the scientific community, it becomes difficult, sometimes impossible, to reproduce the analysis pipeline and replicate results (Munafo et al., 2017). The present work stresses the need for a shift in the philosophical framework driving data analysis in cognitive science, which is opening a window of plausible results instead of accepting a unique (often unsatisfactory and reductive) conclusion drafted on an unthoughtful data analysis (for a debate, see also Scheel et al., 2021).

As psychophysiologists and neuroscientists, we have been persuaded that in neuro and psychological sciences, we *find*, *collect*, and *observe* data. Nevertheless, we commonly build and shape datasets as a function of specific analysis (Del Giudice & Gangestad, 2021). Posing our attention to the proposed field of interest, it is well known that infants’ data shows a higher intra- than inter-individual variability across a wide range of cognitive abilities compared with data from the adult population (for a debate, see Siegler, 2002). However, traditional analyses like repeated measures ANOVAs are commonly conducted on aggregated data (i.e., average pupil size per participant and condition for the entire trial), whereas mixed-effects regression would be the appropriate methodology on individual trials (Brysbaert & Stevens, 2018; see also Mathot & Vilotijević, 2022). Moreover, with repeated measures ANOVAs the violation of the statistical assumptions, e.g., sphericity, increases the likelihood of obtaining false positive results (for a debate see Boisgontiera & Cheval, 2016). That is, cognitive scientists have often been involved in developing theories starting from the interpretation of results framed in those statistical approaches that do not efficiently deal with trial-by-trial and individual variability (see Card, 2017).

Indeed, cognitive scientists encounter a number of degrees of freedom that do not directly reflect data *per se* but more often reflect a byproduct of data processing that hides several degrees of uncertainty (Simmons et al., 2011, Wicherts et al., 2016). In other words, the methodological and analytical multiverse has always been present in cognitive science. However, the issue of building datasets has also been hidden by problematic “risk-permeable” research practices that, although being relatively rare in infancy research (see Eason et al., 2017), may threaten data integrity. Among many candidate tools to stem any replicability and reproducibility crisis, some authors have proposed the multiverse approach as a priming philosophical framework for data analysis. The multiverse approach is a philosophy of statistical reporting of the results of many plausible statistical analyses showing how robust the findings are (Dragicevic et al., 2019). It shows the robustness of a data collection across several steps of data processing (Steegen et al., 2016). In other words, the leading question is not only limited to finding statistically significant results, but rather the investigation of whether the estimated effects are robust or driven by data processing.

In the present study, we dealt with a possible ‘garden of forking paths’ (Gelman and Loken, 2014) offered by psychophysiology applied in infancy research. Importantly, the present work also adopts an approach to infant pupillometry that estimates the effect under investigation while dealing with individual variability, that is, including both fixed and random effects in statistical models. We hope our simple (though not trivial) empirical illustration helps developmental scientists to adopt, implement, and visualize the multiverse of results resulting from a single data collection. Of note, the following illustrative example is accompanied by open-source R code.

### An illustrative example: The case of pupillometry in developmental science

The study of developmental cognition in preverbal infants faces several challenges, and it is highly constrained by the use of indirect methods. In fact, young infants cannot follow any verbal instruction and have reduced control of their own body, even if they are active learners since the neonatal period. Thankfully, scientists have developed a number of measures to gain insight into infant cognition, with looking times at different stimuli being among the most common measures (e.g., Aslin, 2007; Gredebäck et al, 2009; Oakes, 2012; Santolin et al., 2021). Nevertheless, looking times easily decrease over time regardless of the task, making it difficult to disentangle their interpretation (Jackson & Sirois, 2009; see also Sirois & Jackson, 2012). In contrast, pupil variations are considerably less affected by fatigue during trials because only a few seconds of exposure to the stimulus are enough to detect attention fluctuations locked to a specific event (for a review see Hepach & Westermann, 2016). This makes pupillometry a powerful tool in infancy research. However, for the sake of completeness, despite the many advantages introduced by pupillometry, the artifacts and sources of noise that can alter the recorded signal are significantly greater compared to the collection of eye movement-related measures. Therefore, we strongly suggest implementing both measures in a complementary manner in studies with developmental populations. Indeed, pupil dilation and constrictions depend mainly on the variation of distance and luminance of the stimuli with respect to the observer (Mathot & Vilotijević, 2022), and infants and children are usually more inclines in actively exploring their surroundings and less incline in following precise instructions than adults during data collection. However, it is possible to investigate psychological processes, such as attention, arousal, and cognitive load by controlling for it (Beatty, 1982; Karatekin et al, 2004; Porter et al, 2007), as it has been shown across numerous studies conducted with adult and infant populations (Hepach & Westermann, 2016; Laeng et al., 2012).

According to classical theories, in early childhood, participants familiarize themselves with a stimulus when the autonomic nervous system's response to repeatedly presented stimuli decreases over time (Sokolov, 1969; Colombo & Mitchell, 2009). In the specific case of the present study presented as an illustrative example, it is expected that the pupillary dilation response (which is an index of sympathetic activity) will decrease over each trial and be reduced in the last trial compared to the first. This reduction in pupillary dilation response over trial time and thus over the experiment's time should indicate that the information, i.e., the object, has previously been processed and recorded in memory (familiarized) so as not to be evaluated as a new stimulus by the cognitive system. In particular, the present study compared the impact of audiovisual vs*.* visual stimuli familiarization in 12-month-old infants. Regarding the specific effects of audiovisual versus visual stimulation, it is indeed important to consider whether any observed differences may be due to better familiarization or alternatively to increased boredom. It has indeed been suggested that pupil size tends to decrease over the course of an experiment, which can be attributed to factors such as time on task and boredom. In particular, tonic pupillary changes are especially evident in situations of fatigue, when pupil dilation variability augments and its size diminishes steadily (Karatekin, 2007; McLaughlin et al., 2023). This is an interesting question that requires careful consideration in infancy research. It is possible that some stimuli may capture infants' attention more effectively or enhance their engagement compared to other visual stimuli, leading to better familiarization and potentially reducing boredom-related effects. However, it is difficult to clearly disentangle the two constructs, given that both boring and familiarized objects are expected to elicit a reduced pupillary response (Chen & Westermann, 2018). Overall, it is crucial for developmental scientists in the infant research field to carefully consider and address potential confounds such as time on task effects and boredom when considering pupillary responses and other behavioral observations.

It is essential to note that in this study focused on the multiverse analysis approach applied to cognitive pupillometry in infancy research, the statistical sample analyzed is very small (*N* =16), unfortunately representing the scarcity of large samples in developmental science (Frank et al., 2017). In general, to ensure that the statistical results are representative of the population to which they are assumed to generalize, it is good practice to conduct *a priori* power analysis, that is, a precise hypothesis about the expected effect size and a fine computing of the adequate sample size, before the data is collected. This caution allows for the best use of statistical inference, ensuring predictability and replicability of the data (Fiedler, 2017). Given the illustrative purpose of this study, it is important to note to the reader that the sample used is solely for convenience and therefore, it is not possible to define whether this data is useful for theoretical advancement. However, their usefulness and informativity remains and helps to promote methodological advancements in preprocessing and modeling of infant pupillometric data.

Importantly, just like most psychophysiological measures, the richness of pupillometry datasets can be very useful in testing sophisticated hypotheses, it also creates many opportunities to obtain effects that are statistically significant but do not reflect true differences among groups or conditions (*bogus effects*) (see also Luck & Gaspelin, 2017). The main objective of the present work is to discuss the robustness of the results offered by cognitive pupillometry applied to infancy research, and their degree of dependency on processing and analytical decisions, which is the nuance and the limits of cognitive pupillometry in developmental science. In doing so, the present contribution aims at increasing reliability in developmental science by focusing on the robustness of results (for a debate see also Byers-Heinlein et al 2021; Frank et al., 2017). We did so by adopting an explorative approach by means of both (a) a multiverse of datasets and (b) a multiverse of modeling that can be applied at specific steps of pupillometry processing. Specifically, we applied a range of possible choices that allowed us to explore the methodological multiverse, whereas the analytical multiverse allowed further exploration of the robustness of the results across the multiverse of datasets.

## Method

### Participants

We recruited participants from a database of Italian newborns available in the Department of Developmental and Social Psychology, University of Padova. Research was conducted in accordance with the Declaration of Helsinki. Parents provided their informed written consent. The research protocol was approved by the Ethics Committee of our University. Among the 34 12-month-olds who participated in the study (SD = .84, 15 girls), we focused on 16 infants (M = 11.9 months, SD = .9, five girls) that completed the whole task (audiovisual and visual block).

**Fig. 1:**
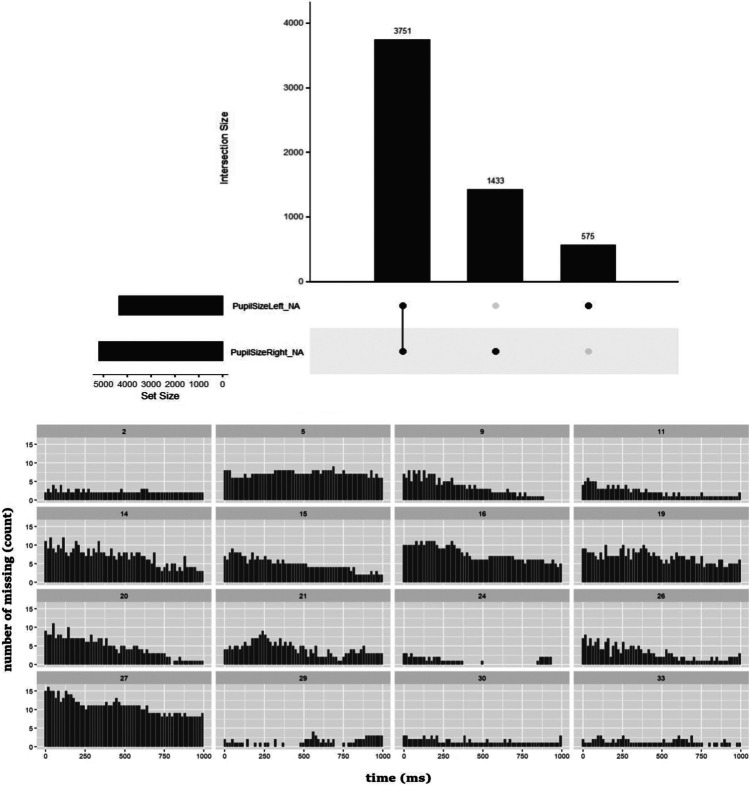
Intersection of missing data patterns between eyes (left and right eye) the three columns represent a different combination of the two eyes with missing responses (i.e., those with black marks). Missing data patterns are also shown by participants (id: identification number) in the whole experiment time window (time in ms)

We obtained 16,041 valid measurements, whereas we discarded 3751 missing data points, representing 23% of the whole time series data. Figure 1 (upper panel) shows missing data that were set to NAN specifically to avoid distorting the data and rendering the analysis invalid. Notably, missing values are ubiquitous in infancy research, Fig. 1 (lower panel) shows a visual inspection of trackloss across time by participants. Such a sanity check of missing data might be a potentially best practice that offer insights into individual differences shown in cognitive pupillometry applied to infancy research.

### Apparatus

Visual stimuli were presented with the Open Sesame software version 3.1 (Mathôt et al., 2012) on a 27-inch monitor. A remote, infrared eye-tracking camera (Tobii X2-60 Eye-Tracker) placed directly below the screen recorded the participant's eye movements using bright pupil technology at a sampling frequency of 60 Hz. The audio stimuli were presented with two speakers (KRK rokit rp 5) placed on the right and left of the screen. The experimental session took place in a room with semi-darkness constant luminance guaranteed by a lamp positioned 1 m away behind the participant. The room presented a dark curtain that isolated the participant area from the experimenter area.

### Stimuli

#### Visual stimuli

Visual objects used in both the familiarization phase and the overlap task were selected from the Novel Objects Unusual Noun (NOUN) database (Horst & Hout, 2016). For each object, NOUN provides measures of familiarity (i.e., the percentage of adults that reported to have already seen the object), nameability (i.e., the percentage of adults who named the object with the same name) and color saliency (i.e., the percentage of adults who spontaneously referred to the objects' color(s) when asked to name the object). We used two objects that were expected to be unfamiliar to our participants ('object 2016', familiarity score = 28%, name-ability score = 21%, color saliency = 61%; object 2025, familiarity score = 6%, name-ability score = 14%, color saliency = 58%). All stimuli were equated in terms of luminance and color using LightRoom software and GIMP2 to avoid any luminance confounding effect. Stimuli (and measures) are listed in the open repository.

#### Auditory stimuli

Linguistic sounds (audiovisual stimuli) were composed of two disyllabic pseudowords selected from the NOUN database: /coba/ and /dupe/. These pseudo-words are phonotactically legal in Italian and have the most common syllabic structure in the infants' native language (i.e., the consonant-vowel (CV) sequence with a trochaic stress pattern). Stimuli were recorded with the Audacity software (equipment: SHURE PG58 microphone and M-AUDIO Fast Track). The audio stimuli were recorded by a female speaker chosen from three different recorded voices because this resulted in a qualitatively stable spectrogram. The auditory stimuli were then matched in terms of intensity and pitch (see Plot of spectrogram in the supplementary materials). The two stimuli had a similar duration (521 ms for 'coba' and 534 ms for ‘dupe’). Stimuli are available in the open repository.

### Procedure

Before the experiment started, we welcomed the parents and infants to the lab so that they could feel comfortable in the environment. Then, participants sat in an infant highchair, with parents standing behind the infant's seat 60 cm away from a 27-inch screen 109 pixels per inch. At this point, a five-point calibration procedure started (top-left, top-right, center, bottom-left, and bottom-right).

The study had a fixed factor familiarization block (2: Visual vs. Audiovisual) in a within-participant design (all participants were exposed to both familiarizations with visual and audiovisual stimuli). We recorded infants' eyes movements and pupil dilation as response variables during the two familiarization blocks. Only when the eye-tracker reached adequate calibration fit, the experiment started with one of the two familiarization blocks (audiovisual vs. visual), randomly between participants.

**Fig. 2:**
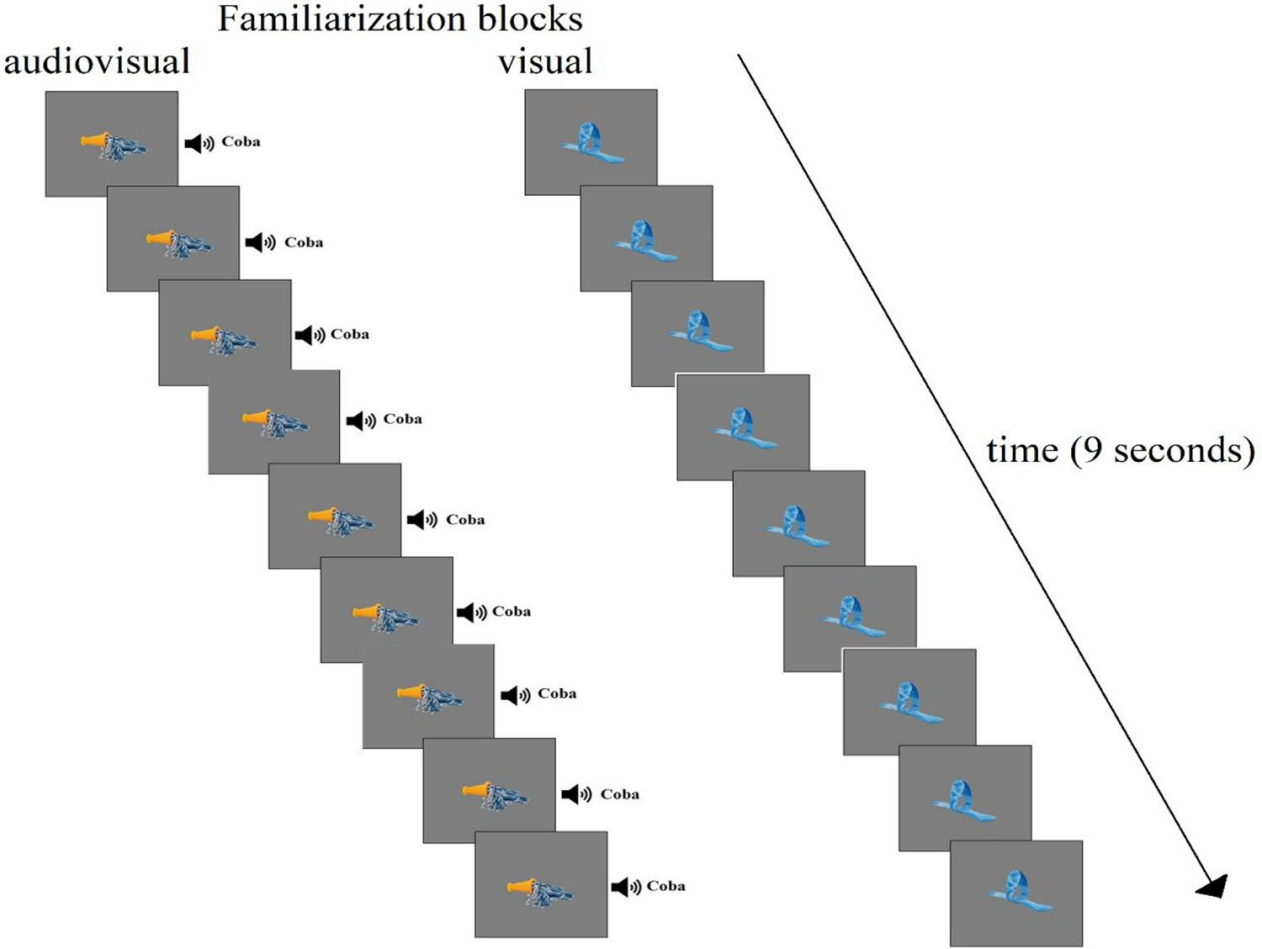
Participants performed two separate familiarization blocks consisting of nine trials (1 s each). A trial started when the eye tracker reached 100 ms of gaze points at the central visual stimulus; only in the audiovisual familiarization after 100 ms from the stimulus onset the audio started. Note that audio and visual stimuli were counterbalanced among blocks and participants

Each familiarization block started with the appearance of a static visual object. Participants saw one different object in each block. Visual objects were counterbalanced among participants and blocks. Objects were presented at the center of the screen (10° x 10° in visual degrees). In the audiovisual familiarization block, the auditory stimulus started when the participant reached 100 ms looking at the visual object (contingency procedure). In the visual familiarization block, a visual object was presented without any auditory stimulation. Each block consisted of nine trials (1 s each), as shown in Fig. 2. Each trial lasted 1000 ms and was presented in sequence with no pause between trials within the block. Note that the choice to place the trials within each block, one after another, allows for the exclusion of luminosity excursions between trials. It should be noted that this is particularly relevant for the audiovisual block, as it allows for relating the pupillary response with the presentation of the auditory stimulus in the audiovisual familiarization block. Of course, the subdivision into trials is purely methodological, as can be inferred from Fig. 2, in fact a participant can experience the 9-s familiarization period for each block as a single repetitive event. This approach allows for a detailed study of the familiarization processes over time by capitalizing on pupillometry (Colombo & Mitchel, 2009).

## Data analysis and results

### Degrees of freedom in pupil data management and modeling

The diameter of the pupil was continuously traced during the two familiarization blocks. Pupil size variability consists of a tonic state and a phasic response, and we evaluated only the latter. Pupil data were analyzed along with the whole trial window. To obtain a measure of the phasic response, we calculated the average of raw pupil diameter values from the two eyes when the eye tracker got a good signal from both eyes. Otherwise, measurements where only one eye was tracked (see Fig. 1) they were either excluded or interpolated (see *Degree of freedom #3: Dealing with blinks)*.

### Data processing: Building a multiverse of datasets

#### Degree of freedom #1: Extreme yet plausible values

**Fig. 3:**
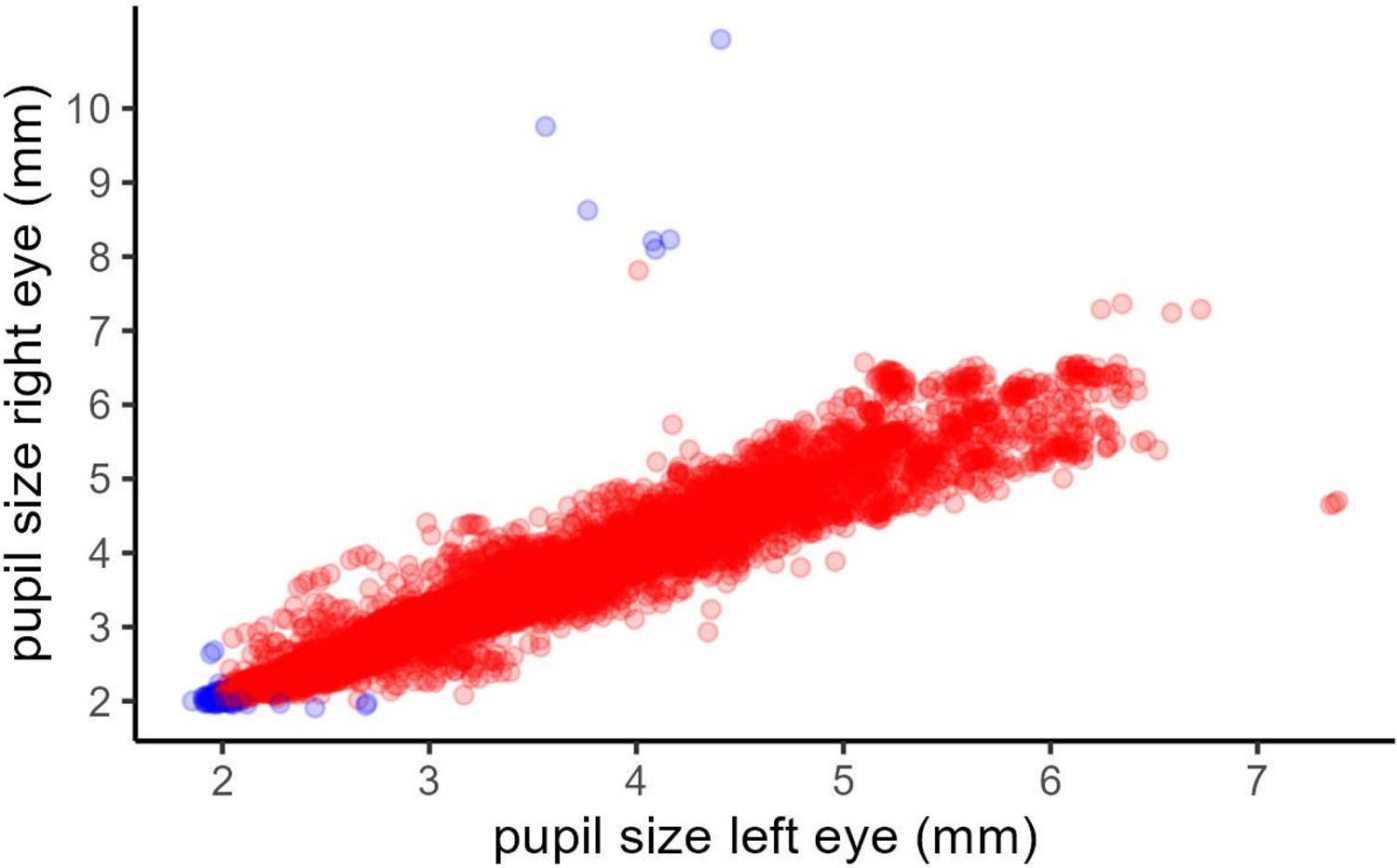
Scatter plot correlating left and right eye’s pupil size. *Blue points* indicate the values excluded in the second filtered dataset (trimmed dataset)

We started our data processing by looking at the pupil size data traced by the eye tracker. Figure 3 shows a basic scatter plot depicting the *X* and *Y* coordinates of gaze points plotted across the whole screen space. As cut-off values are usually applied accounting for human physiology (e.g., Mathôt et al., 2018), we moved a first step into the multiverse of data processing by building an alternative dataset only including pupil size values higher than 2 mm and lower than 8 mm (step 1: filtered vs. unfiltered data), while keeping the full dataset into consideration. This step allowed us to check to what extent extreme yet plausible values introduced substantial variability to the data, possibly driving the results interpretation at both the trial and the subject level (Mathôt et al., 2018). Moreover, the impact of the variability introduced by the extreme yet plausible values adds fundamental knowledge on the robustness of the effects under scrutiny as it has been traditionally investigated as a crucial preliminary step in statistical analysis (for a debate see Reiss et al., 1997). Finally, as a sanity check, we looked at the degree of correlation between the two eyes (Pearson’s *r* = .96). Such correlation is expected to be very close to 1, based on typical human physiology.

#### Degree of freedom #2: Area of interest

**Fig. 4:**
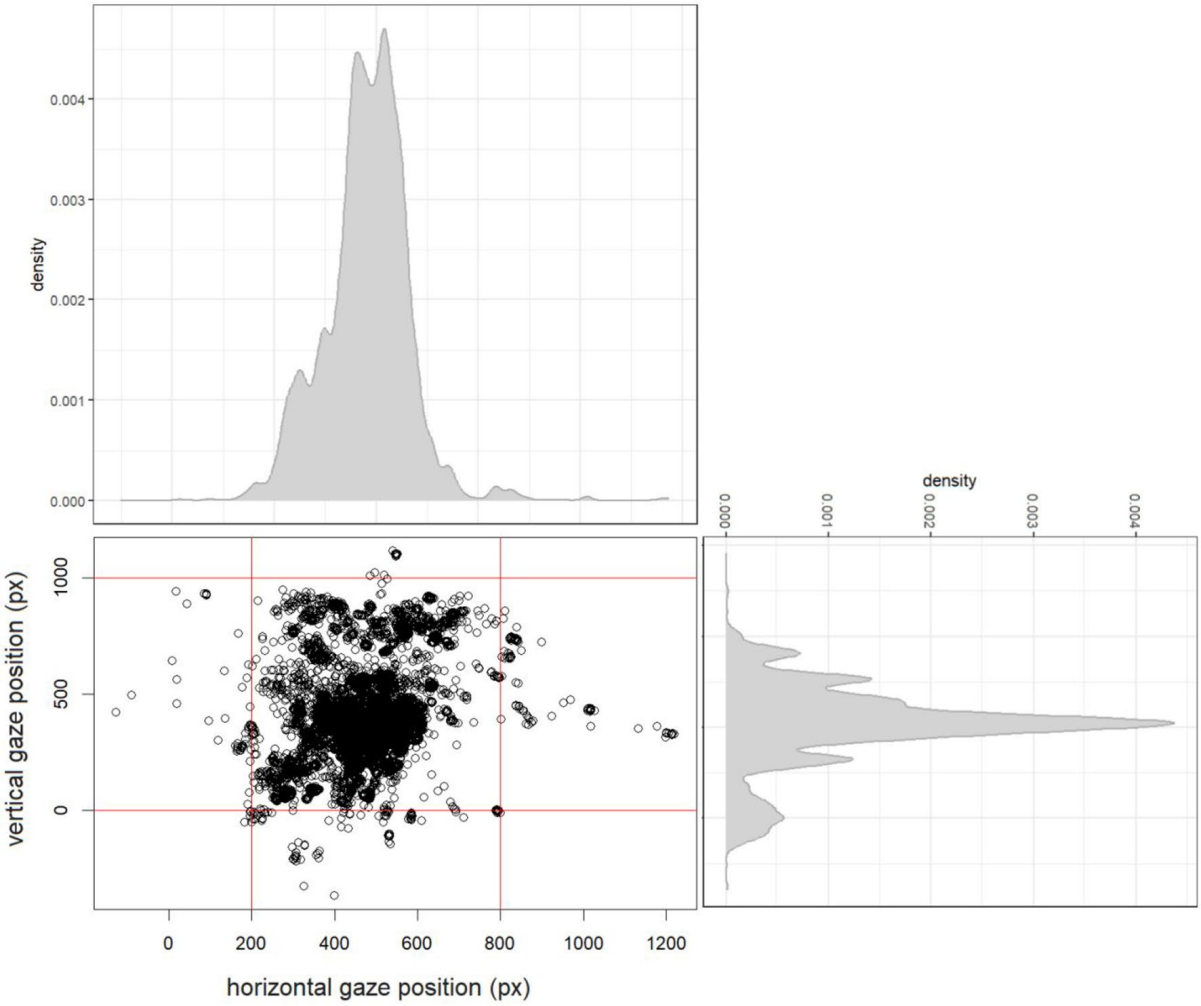
Gaze-points coordinates, each corresponding to a pupil size value. The *vertical* and *horizontal red lines* indicate the AoI of interest under scrutiny, i.e., the central red rectangle. Density plots of the GazePoint *X* and *Y* in arbitrary units

In the previous step of the methodological multiverse, we obtained two datasets starting from the same data collection. We then moved a second step deeper into the methodological multiverse by focusing on gaze data, and specifically by building two datasets based on the area of interest (AoI, including the visual stimulus and a margin of 1 cm) (step 2: whole screen vs. AoI). Importantly, in this illustrative example the manipulation aimed at detecting the impact of centered visual stimuli on pupil size variations. Therefore, the objective of this step was to estimate the variation in pupil size measured as a function of visual stimuli presented at the center of the screen. That is, when participants looked directly at the centered visual stimuli, the pupil was recorded as a near-perfect circle. However, participants made several eye movements around the AoI, implying eye rotation, and possibly leading to measurement error. Figure 4 shows all gaze data mapped into a 2D coordinate system (*X* and *Y*) corresponding to the whole eye-tracked space. It is fundamental to outline here that such a sanity check offers insightful consideration on the efficiency of a given paradigm, especially in those that implement novel and original procedures. Specifically, Fig. 4 clearly shows that our procedure captured infants' attention towards the objects, as indicated by the majority of data points falling within the AoI. This is particularly interesting in the context of the present study given that in the audiovisual familiarization the audio could be still active also when infants were looking near the AoI. For instance, the clusters of data at the AoI’s borderlines might have nothing to do with the research question, yet it is completely arbitrary to eliminate them given that excluding data points outside the AoI may be informative of the impact of audiovisual stimuli which do not need to be looked at to be processed, on attention.

As a corollary, we built two additional datasets including and excluding data points falling outside the central AoI. In doing so, we added a forking path to our multiverse analysis, and we obtained four working datasets (i.e., two from extreme yet plausible values and two from AoI-related data processing) coming from the same data collection (step 2: whole screen vs. AoI).

#### Degree of freedom #3: Dealing with blinks

The analysis of blink data is of methodological importance in eye-tracking research, particularly when measuring pupil changes over time with blinking being a physiological process that affects the measurement of pupil size (Mathot & Vilotijević, 2022). Therefore, treating blink data can enhance the quality of data, improve the reliability and validity of results, and provide a more accurate understanding of the cognitive processes involved in object perception. However, when dealing with blink data in eye-tracking research, researchers face the dilemma of either excluding vs. interpolating missing data caused by blinks. Both approaches have their advantages and disadvantages. Excluding blink data can reduce the risk of introducing artificial changes in the pupil size measurements but can also lead to a loss of valuable information. On the other hand, interpolating missing data can preserve the temporal continuity of the data but may introduce catastrophic noise or distortion in the signal (Mathôt et al., 2013). In summary, the decision to exclude or interpolate blink data ultimately depends on the researcher and the specific characteristics of the data, offering the opportunity to explore a further forking path in the pupillometry multiverse.

Here, starting from the four datasets previously built from a single data collection, we created eight datasets, half of the datasets have blink exclusion and the other half has blink interpolation (step 3: no blinks vs. interpolated blinks). In particular, we used the ‘na_interpolation()’ function in R to fill in missing data in a vector. When a vector contains missing values (represented by NA), it can create problems when performing data analysis or visualization. The na_interpolation() function uses various interpolation methods such as linear, spline, and polynomial to estimate the missing values. The method used is determined by the method parameter, which can be set to "linear", "spline", or "poly". By default, the method parameter is set to "linear". Linear interpolation estimates missing values by drawing a straight line between two neighboring data points. Spline interpolation estimates missing values by fitting a smooth curve between the neighboring data points. Polynomial interpolation estimates missing values by fitting a polynomial equation to the neighboring data points. Nevertheless, even if we used the linear interpolation, there are several alternatives to interpolate blinks based on observed data that can be included in a multiverse analysis to reduce the impact of specific interpolations on results (see Mathôt, & Vilotijević, 2022).

**Fig. 5:**
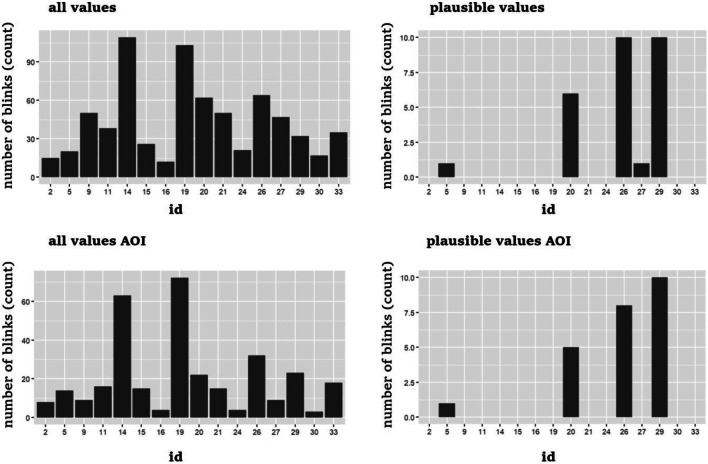
Distribution of blink detected across the participants (id) in the four datasets, i.e., all values in the whole screen, plausible values in the whole screen, all values within the area of interest (AOI), plausible values within the AOI

Figure 5 shows the number of blinks detected for each participant as a function of the two degrees of freedom addressed thus far in the multiverse analysis, i.e., dealing with extreme values and the area of interest. It is important to note that as shown in Fig. 5 the majority of blinks are coupled with the datasets including extreme values of pupillary diameter. This is not surprising, considering that blinks occurring naturally during vision, which serve to hydrate the eye, necessarily produce rapid changes in light flux incident on the retina. Therefore, even if blinks are essential for maintaining the health and lubrication of the eyes, they can introduce variability in the measurement of pupil diameter due to the rapid changes in retinal luminosity caused by eyes closure and reopening. These rapid fluctuations can result in transient changes in pupil size, potentially leading to extreme values. However, blinks have also been interpreted as an indirect measure of reduced attention towards a stimulus, and it has been indicated that adopting an intensive longitudinal approach to study the rate blink rate can lead to finely investigate developmental change associated with attention regulation in the first year of life (Bacher, 2014).

In the context of the present study, the simple description of the distribution of blinks in relation to the preprocessing steps allows us to understand the importance of considering the distribution of blinks when evaluating the robustness of the data in terms of interpolation versus exclusion of blinks. In fact, in datasets containing all values, interpolation necessarily leads to a greater number of observations, increasing the statistical power of subsequent analyses. However, it also increases the probability of imputing data that are unrelated to the measurement of interest (attention toward the stimuli), thereby raising the likelihood of invalidating the estimated measurement.

#### Degree of freedom #4: Baseline correction

As a further step into our methodological multiverse of pupillometry data, we faced baseline correction. A pupillometry analysis with baseline correction implies that pupil sizes are firstly compared with those recorded over a baseline window, nested by trial and participant. Thus, the dependent variable becomes the change in pupil size relative to the mean or median baseline value. Such an approach allows for a within-trial analysis, that is, an analysis in which each trial (nested by subject) is taken into account and considered as a random effect.

However, it is crucial to highlight that baseline correction can sometimes lead to artifacts, that is, data distortion due to measurement error. Artifacts can be detected by including a sanity check comparing baseline-corrected pupil trace and raw (not corrected) data, a particularly important practice for a multiverse framework like the one we presented, in which we admitted more than one plausible level for baseline correction.

**Fig. 6:**
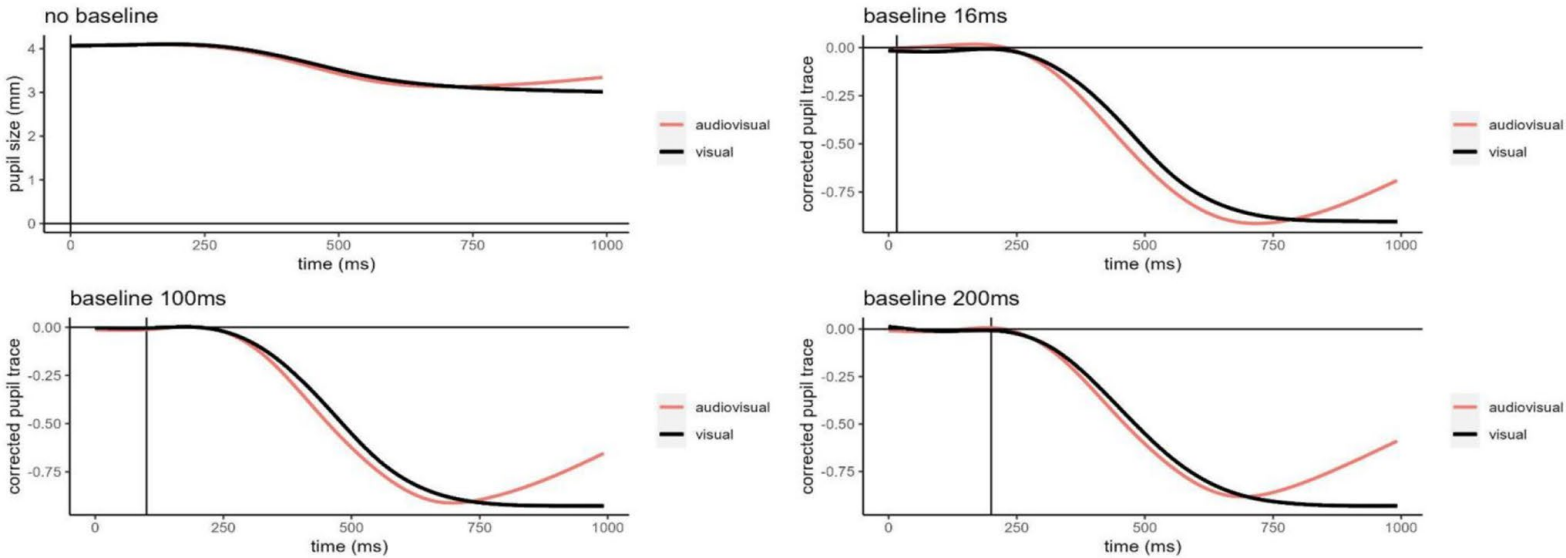
Average pupil size variation (no baseline) and pupil changes relative to baseline (16, 100, and 200 ms) smoothed across time, we used the dataset with trimmed values filtered by the AoI and interpolated blinks for illustrative purposes. The *red* and *black lines* represent the audiovisual and visual familiarization, respectively. The *vertical line* indicates the end of the baseline (when present)

To reduce the multiverse space, we decided to follow Mathôt et al. (2018)’s recommendation to prefer a subtractive baseline correction on a trial basis (pupil = trial pupil size – baseline) instead of a divisive baseline correction (pupil = average/baseline). Indeed, it has been suggested that the latter should distort the data compared to the former (Mathôt et al. 2018). Here, we corrected our data with a subtractive baseline method. Specifically, we choose three plausible baseline interval lengths for illustrative purposes, that is, a short, a medium, and a long baseline corresponding to the median pupil dilation value of the first ~16, 100, and 200 ms after the stimulus onset, respectively. Each of these three plausible baselines were separately subtracted by each trial within each participant, and across the two familiarization blocks. Figure 6 shows an example of pupil size variation and pupil size change relative to baselines over time, in the dataset with trimmed values only, filtered by the AoI and with interpolated blinks.

Note that by selecting a subtractive baseline we adopted arbitrary choices adding constraints to our multiverse of possible results. Starting with four datasets resulting from the previous steps of the data processing, we applied the three baseline correction procedures to each of them, hence obtaining 24 plausible datasets from a single data collection (step 4: 16-ms vs. 100-ms vs. 200-ms baseline correction).

Figure 6 shows an example of how baseline correction modifies the pattern of pupil size over time, compared with no baseline correction. In particular, all the three baselines (step 4) steepened the pupil curve slope, showing a substantial restriction of pupil across time, compared with the pupil size variation with no baseline correction. However, the changes in pupil size as a function of time showed to emerge slowly (i.e., > 200-ms manipulation onset) suggesting that baseline correction did not introduce influential artifacts (Mathôt et al., 2018). Of note, the rationale behind the inclusion of a longer baseline relies on the fact that any difference in attention deployment due to the presentation of visual vs. audiovisual stimuli should emerge after the critical latency period (200 ms), which is typical of the pupil dilation response to cognitive rather than physical (e.g., light) factors. Notably, baseline correction in cognitive pupillometry deals with the high variability shown by infants. Therefore, the multiverse of baseline corrections takes into account this variability increasing the results robustness.

#### Degree of freedom #5: Participants inclusion

The exclusion of missing data so far has concerned the minimum units of the experiment, that is, individual observations (i.e., step 1, step 2, and step 3). However, the impact on the statistical results led by the presence of missing data may depend on the amount of missing data and on the mechanism generating the missing data (Bennett, 2001) producing a dataset that can be imbalanced with respect to covariates of interest. As the final crossroad in this multiverse analysis, we will attempt to exclude or include those participants who generally exhibit 30% missing data in total during the trial recording. This will allow us to estimate the degree of robustness of the results excluding these participants, according to the multiverse of other plausible scenarios. By doing so, we can add a brick into the wall of a more detailed representation of the experiment's findings.

**Fig. 7:**
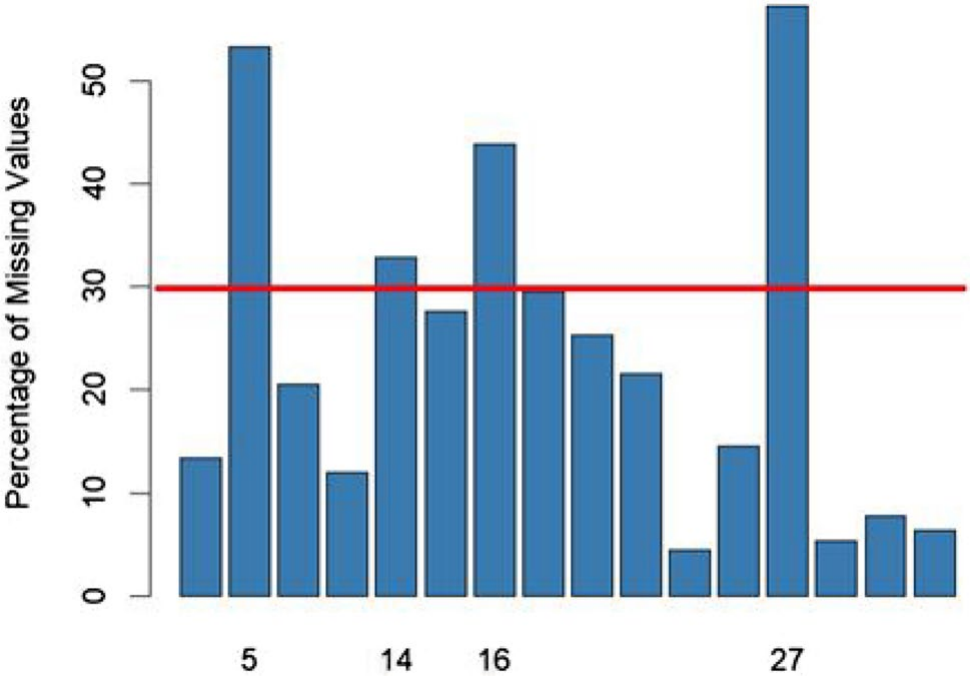
Percentage of missing values by subject (ID). The *red line* indicates the cut-off value. Note that all that only ID participants above the cut-off are shown in the *x*-axis

Figure 7 shows the percentage of missing data by participant, showing that participant ID 5, 14, 16, and 27, have more than 30% of missing data, compared with the group. Thus, we explore the last part of our multiverse by including and excluding those participants from the final analysis (step 5: inclusion vs. exclusion of participants).

#### Degree of freedom #6 A multiverse of models

Our example suggests that attention deployment is likely to have a nonlinear relationship with the time course of both familiarization procedures (Fig. 6, see also Hershman et al., 2022; Wass et al., 2016). Thus, as a last step into the multiverse, we modeled both the time-course effect of familiarization and the mean effect of familiarization block on pupil changes. That is, we explored the impact of including (vs*.* excluding) the interaction between familiarization block (i.e., audiovisual and visual) and time (as a continuous predictor, in ms). In so doing, we showed by means of an illustrative example whether and how smoothing time increased the plausibility of pupil dilation statistical modeling within each of the 48 datasets that we built.

Specifically, we capitalized on generalized additive mixed modeling (GAMM, Wood, 2011) for the analysis of pupillary data. A GAMM is a statistical model that combines the flexibility of generalized additive models (GAMs) with the ability to account for random effects in mixed effects models. GAMMs allow for the analysis of complex, nonlinear relationships between dependent and independent variables, by means of smooth functions including both continuous and categorical predictors, while accounting for the correlation among observations within clusters or groups. The random effects component enables the inclusion of hierarchical structures, such as nested or repeated measures, within the data. This makes GAMMs useful for modeling a wide range of data types, including time series, spatial, and longitudinal data. The model is estimated using penalized regression techniques, which help to avoid overfitting and produce more reliable predictions.

A discussion of the technical aspects of smooth functions is beyond the scope of this article (see Wood, 2017), but readers should at least notice that a smooth function can be thought of as a continuous change in pupil size over time. GAMM approximates smooth functions as a weighted sum of a set of base functions to fit the pattern of the data (see Wood, 2017). To clarify the structure of the models, we provided both a formal description and the R code to run the model. All models were fitted with the *bam()* function of the mgcv R package version 1.8-38 (Wood, 2011) in an R environment (Team R. C., 2018). We started with a time model including the interaction between the covariate Time, representing the time in the trial aligned with the onset of the visual stimulus, and the familiarization block, which is a two-level categorical predictor. This model estimated two regression lines over time, one for each level of familiarization block. Then, we specified a simpler no time model that only estimated the mean effects of the familiarization block on changes in pupil size variations. The model included a random effect (smoother term) for the levels of the familiarization block by participant.

In particular, the time model was specified with a fixed factor familiarization block, the smoother interaction terms between familiarization block and time, and between time and participants, as following:

$$Y=\alpha +\beta X+\mathrm{g}1\left(t,X\right)+\mathrm{g}2\left(t,\mathrm{id}\right)+\varepsilon,$$

where Y is the dependent variable, α is the intercept, β is the coefficients related to the familiarization block X (audiovisual vs. audiovisual), g1() defines a smooth interaction function between time and familiarization type, g2() defines two smoothing functions related random effects of time. Those latter terms indicate that for each level of familiarization type, a different non-linear regression line is fitted over Time (i.e., in R parse pseudocode: dependent variable ~ familiarization block + s(time, by = familiarization block, k = 20) + s(time, id, bs = ‘fs’)).

The no time model was specified with the fixed factor familiarization block; a smoother term of familiarization block by participant, as following:

$$Y=\alpha +\beta X+\mathrm{g}1\left(X,\mathrm{id}\right)+\varepsilon,$$

where Y is the dependent variable, α is the intercept, β is the coefficient related to the familiarization block X, g1() defines a smoothing function related to the random effect of the familiarization block, by subjects. (i.e., in R parse pseudocode: dependent variable ~ familiarization block + s(familiarization block, id, bs = ‘fs’)).

**Fig. 8:**
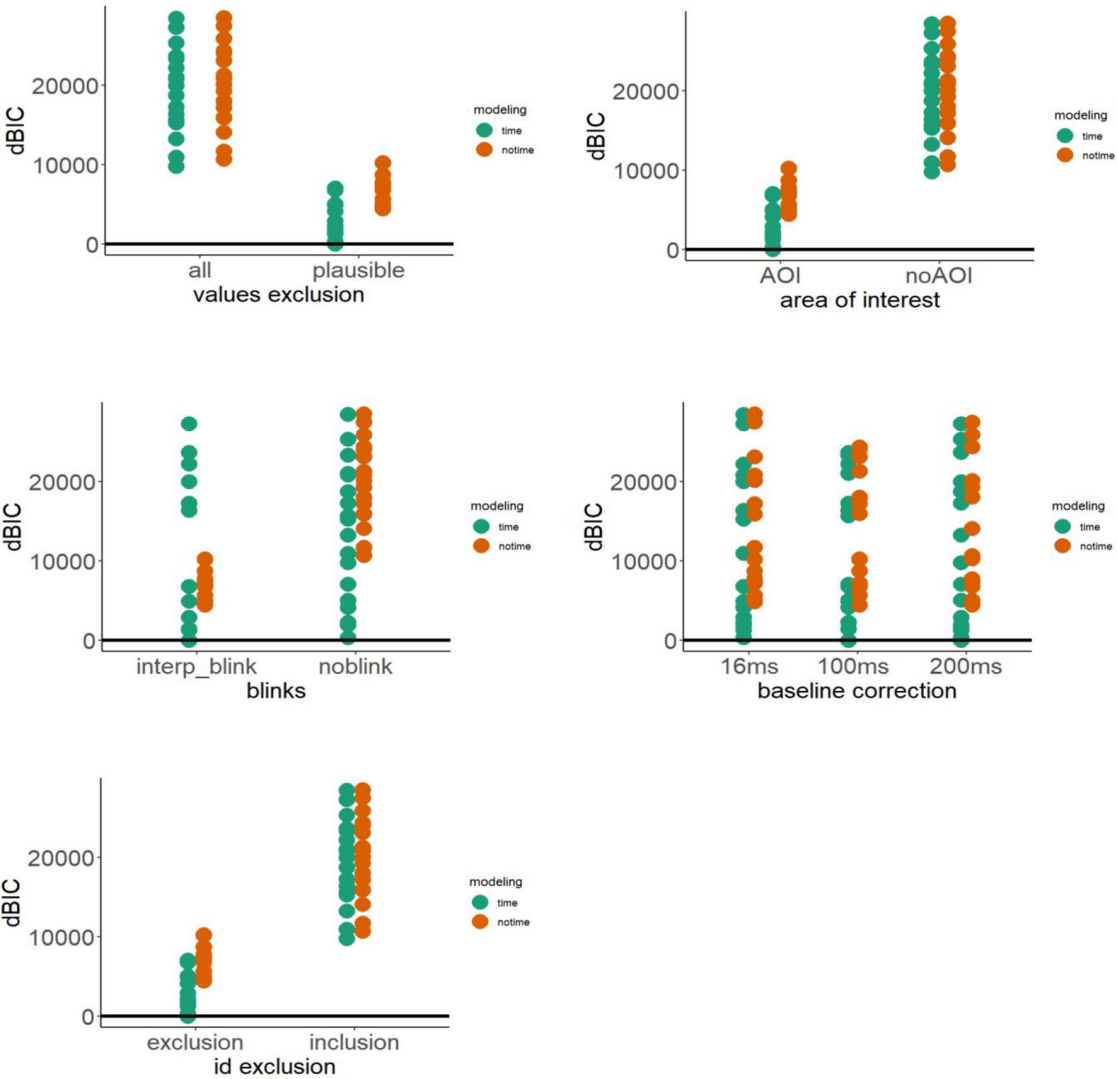

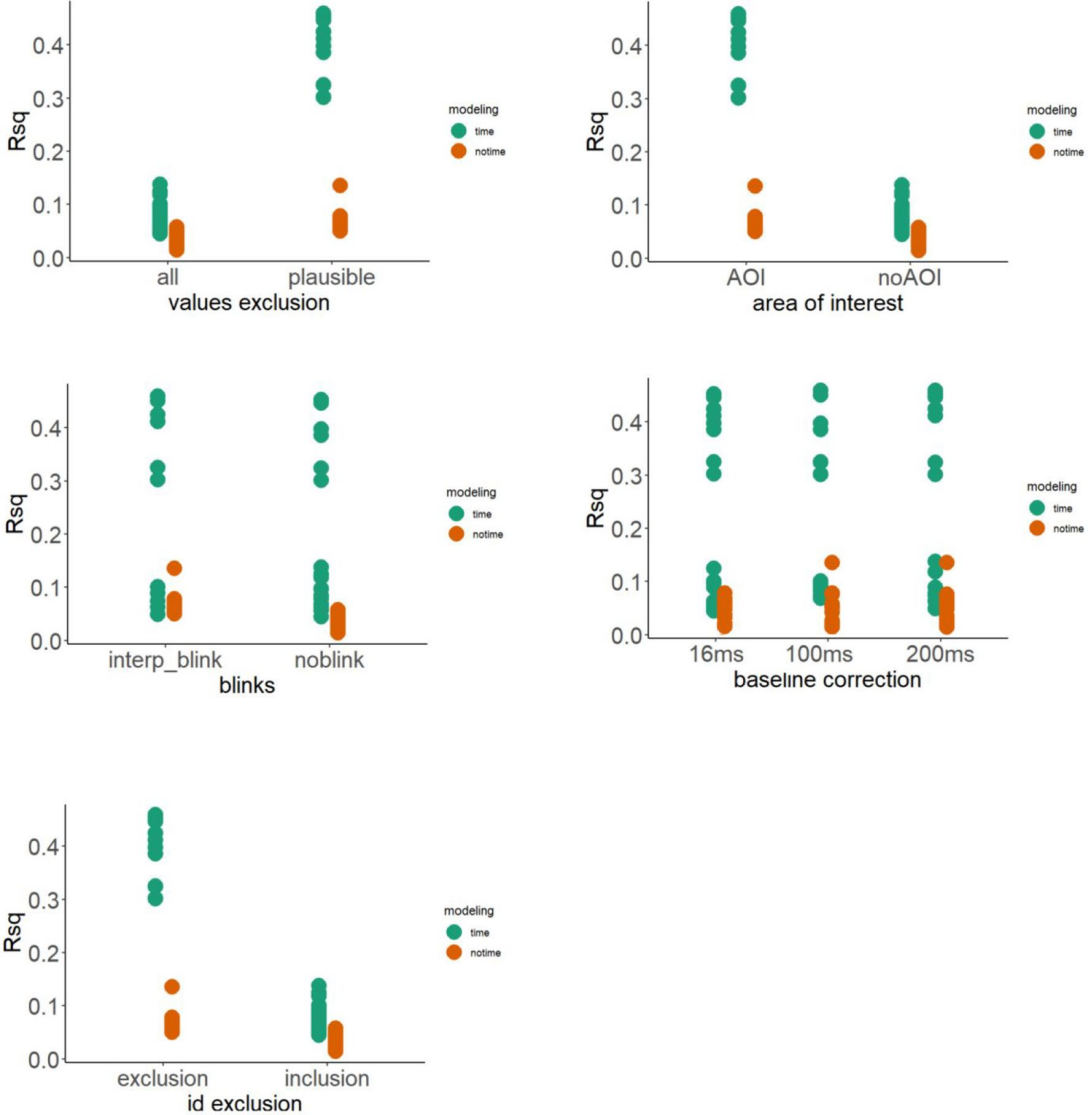
The figure shows the delta BIC (the lower the better) and *R* squared of the two models (i.e., with and without smoother terms for time) for the 48 datasets. Plots are split by the first (extreme vs. trimmed values), the second (no AoI vs. AoI), the third (no blink vs. interpolated blink), the fourth (16-, 100-, and 200-ms baseline) and the fifth (participant inclusion and exclusion) degrees of freedom

Both the time and the no time model were fitted to each of the 48 plausible datasets (step 1 × step 2 × step 3 × step 4 × step 5). The most plausible model was selected following two rationales. First, the best-fitting model was selected using the Bayesian information criterion (BIC) (Raftery, 1995; Wagenmakers, 2007), The BIC is a model selection criterion that is based on information theory and is set within a Bayesian framework. It was proposed by Schwarz (1978) and is also known as the Schwarz information criterion and Schwarz Bayesian information criterion. BIC is calculated using the formula:

$$\mathrm{BIC}=-21\left(\widehat{\theta }\right)+k lo\mathrm{g}\left(n\right)$$

where *l(*$\widehat{\theta }$*)* is the maximized value of the log-likelihood function of the model calculated by parameter values $\theta$ that maximize the log-likelihood function, while *k* and *n* are the number of parameters and the sample size, respectively. The best model is the one that provides the minimum BIC (BIC*), and the evidence against a candidate model being the best model is determined by the magnitude of the difference between BIC of the candidate model and BIC*. The interpretation of the magnitude of delta BIC (i.e., the difference between BIC of the candidate model and BIC*) is as follows: less than 2 indicates weak evidence, 2–6 indicates positive evidence, 6–10 indicates strong evidence, and greater than 10 indicates very strong evidence in favor of the BIC* model (Fabozzi et al., 2014; Burnham, & Anderson, 2004). Then, we evaluated the variance explained by each model using the *R*^2^ coefficient. Note that we compared BIC and *R*-squared among the models estimated within the same dataset, as shown in Fig. 8.

We considered trials as the minimal statistical unit, and we set a minimum of 20 knots as the maximum number of turning points to be used during the smoothing process (Baayen et al., 2017). To explore whether experimental manipulation influenced pupil size, we visually inspected the estimated differential effects between familiarization blocks. We used the R package *'itsadug'* (van Rij et al., 2017) for the interpretation and visualization of the statistical analyses (see van Rij et al., 2019) fully available in the open repository. Note that such arbitrary setting of parameters hides several degrees of freedom (and uncertainty) that could expand the present multiverse analysis.

The results suggest that the time model smoothing the interaction between familiarization block and time is the most plausible model, by showing a consistently lower BIC compared to no time model, in all datasets. Moreover, the time model explained a substantially incremental portion of variance compared to the no time model, as shown by the higher *R*-squared in the former compared to the latter model, again across all datasets. These results indicate that the smoother term of familiarization block × time increased the plausibility of the estimated effects.

#### Results inspection: The impact of by-time smoothing

Table 1 in the supplementary materials openly available in the OSF repository (https://osf.io/p8nfh/) shows the hierarchical structure of the multiverse analysis with the associated regression coefficients and 95% CI of the time model and the no time model. Overall, results suggest that the first (step 1: filtered vs. unfiltered data), second (step 2: whole screen vs. AoI) and fifth (step 5: inclusion vs. exclusion participants) degree of freedom had a substantial impact on the statistical results, with trimmed values falling within the AoI of participant with less than 30% of missing data reducing the uncertainty of the estimated effect, as shown in Fig. 8.

**Fig. 9:**
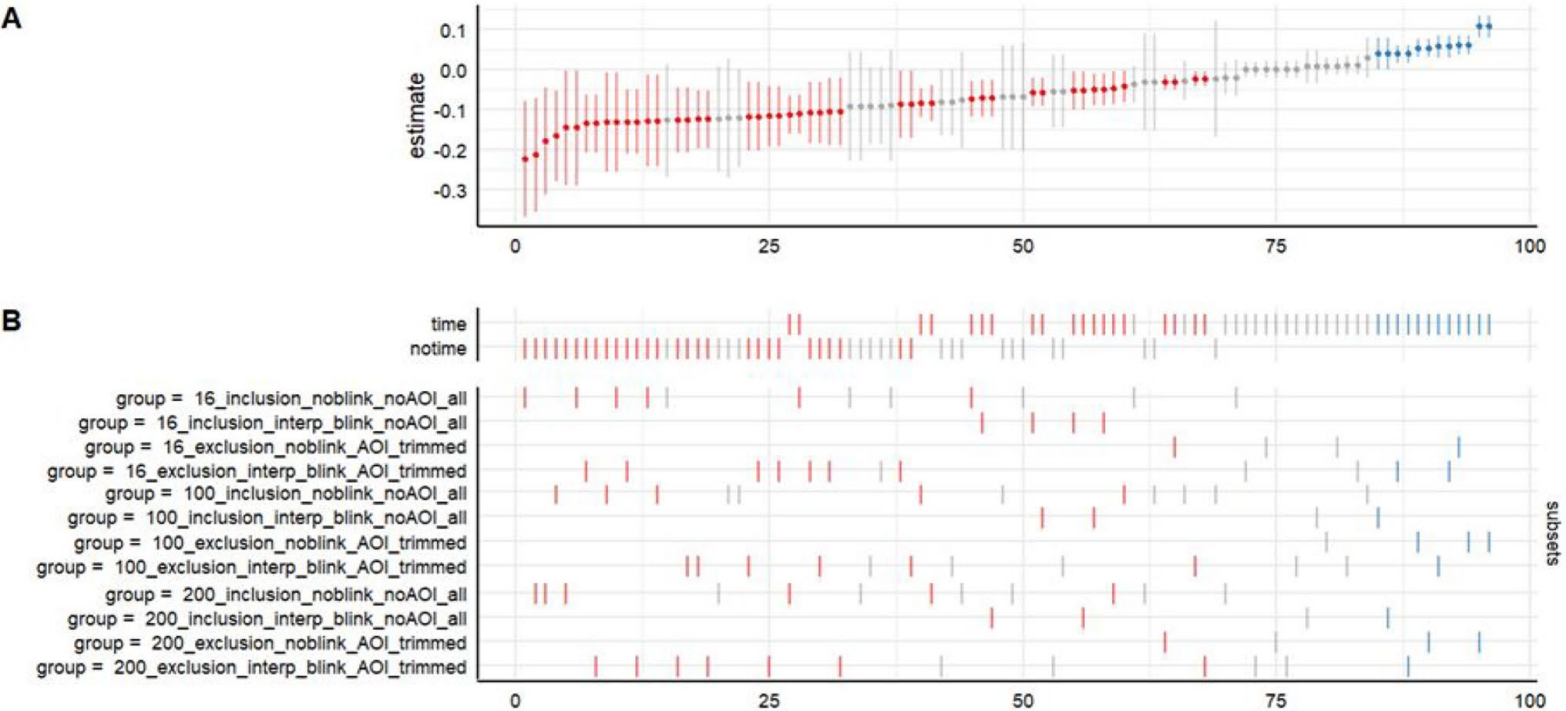
**A** The 96 coefficient’s estimates and relative 95% CI related to the Visual vs. Audiovisual regressor. **B** Relative combinations by the six degrees of freedom of the multiverse analysis. The direction of the significant results are highlighted (negative = *red*, positive = *blue*, *gray* = non-significant). Note that positive estimates (*in blue*) indicate higher pupil dilation for the Audiovisual condition and negative estimates (*in red*) indicate higher pupil dilation for the Visual condition. The *x*-axis represents the model number, while the *y*-axis represents the estimated coefficient

To quantify the robustness of the fixed familiarization block effect (i.e., Visual - Audiovisual block) estimated across a multiverse of analytical choice, we made use of the ‘*specr’* R package (Scharkow, 2019) to plot a specification curve of the results across all specifications of the multiverse, as visual inspection facilitates the selection of plausible statistical results (Simonsohn et al., 2020). Figure 9 shows the specification curve of the 96 estimated effects, that is, Visual - Audiovisual familiarization block effect.

**Fig. 10:**
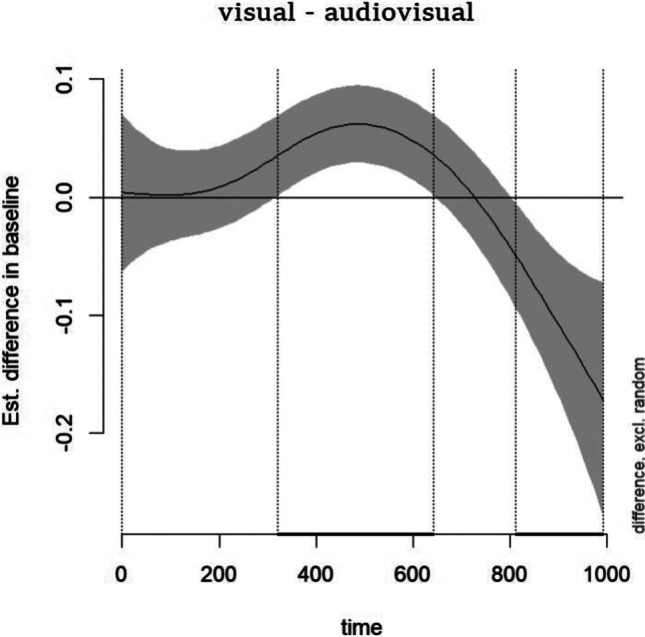
Differential effect plot of pupil changes in the Visual - Audiovisual familiarization block smoothed across time, for the time model on datasets with trimmed values falling within the AoI of participants with less than 30%. The area falling within the *vertical dot lines* indicates the time window in which the differences between conditions were significantly different from 0

Importantly, the effects displayed in the specification curve only show the estimated fixed effects and do not present nonlinear regression lines. Indeed, the smooth functions of the time model cannot be captured by a few coefficients, and a different visualization is necessary for interpreting the nonlinear terms (see Van Rij et al. 2019). As shown in Fig. 10, the significant effect estimated by the time model with the dataset with trimmed values falling within the AoI of participant with less than 30% of missing data indicates that the Visual familiarization induced an early increase and later decrease of pupil dilation, compared to the Audiovisual familiarization block (this emerged across all datasets with trimmed values falling within the AoI of participant with less than 30%; see also supplementary materials).

## Discussion

By taking advantage of pupillometry as an index of familiarization processes, here we stressed the importance of checking the robustness of the results (Weermeijer et al., 2022) to offer a plausible answer to classical investigations in developmental studies with infants. Notably, the present illustrative example used a convenience sample to show a possible way to perform and visualize empirical findings by adopting a multiverse approach to answer fundamental questions in developmental psychophysiology. Specifically, our illustrative example aimed to face uncertainty by checking the robustness in the analysis of pupillometry as an index of attention deployment in infancy. It is important to note that in this study, as in most studies in infant research, the representativeness and generalizability of the results are largely constrained by the number of stimuli used in the experiment (Peterson et al., 2021; Hartshorne et al., 2018). This aspect is critical, albeit informative regarding infants' computational strategies, for the replicability of the results and can only be partially solved by using larger samples and even better cross-cultural and multi-laboratory studies (Li et al., 2022). The main reasons behind the reduced number of stimuli used in infant experiments stem from a behavior of fatigue or fuzziness shown by infants exposed to repeated measures, which can be summarized in the saying *The fun doesn't last forever*. This aspect characterizes the field of developmental cognition and is one among many challenges of research in early childhood and is of course a limitation, it introduces uncertainty that must be declared and accepted. We believe it is important to stress this methodological and practical aspect because it allows us to better quantify and thus address the uncertainty in the interpretation of pupillometric data applied to developmental sciences.

The multiverse of the results that we obtained from a single data collection pointed out several considerations. First, the main result of our analysis indicates that the multiverse approach increases the robustness of the data interpretation by weighting the impact of selected preprocessing choices on the effect under discussion. That is, weighting from the selected arbitrary yet plausible degrees of freedom in cognitive pupillometry, the multiverse offers an increased informativity of cognitive pupillometry, and as a consequence, reinforces the knowledge about infants’ attention deployment during classical familiarization tasks. By means of a multiverse approach, we focused on the robustness of the parameter estimation across a data processing multiverse (i.e., preprocessing degrees of freedom), and as a function of an (illustrative) analytical multiverse.

Nevertheless, it is crucial to note here that we had already constrained the multiverse space even before facing the three degrees of freedom in the preprocessing steps (step 1 × step 2 × step 3 × step 4 × step 5). Indeed, we arbitrarily selected (1) only data by 12-month-olds who completed both familiarizations (*N* = 16/34, possibly implying selection bias) and (2) those timepoints that correctly measured pupil size variation in both eyes. Such decisions signaled two arbitrary and preliminary choices that reduced the multiverse space and its statistical power.

Importantly, such arbitrary choices are not in contrast with a philosophical multiverse perspective. Although all degrees of freedom which might impact statistical analysis should be virtually considered, a full multiverse is often demanding to manage. Nevertheless, it is still worth exploring at least portions of such a multiverse to get more information about the robustness of the effects of interest.

### The impact of the five data processing degrees of freedom: Extreme values, AoI, blinks, baseline correction and participants exclusion

Our results showed that including extreme values (step 1), that is, pupil size outside 2 and 8 mm, had a major impact on results by reducing both the goodness of fit and the explained variance. Furthermore, extreme yet plausible pupil size values produced extreme regression estimates and associated error. Extreme values impacted the estimated effects leading to uncertain functional interpretation of pupil size change as an index of attention deployment (Mathôt et al., 2015, 2017; Laeng et al., 2012). This scenario suggests that reasoning on the impact of extreme yet plausible values of pupil size measurements in controlled experiments with infants can have a dramatic impact on statistical analysis and conclusion of a study (see also Mathot & Vilotijević, 2022). In the specific case of this illustrative example, including extreme values led to the worst fit, less explained variance and higher level of autocorrelation (higher the probability for type I errors). Such a preliminary step of data management is fundamental to build reliable knowledge on pupil size variability in infancy research. In addition, sharing the raw trial-by-trial data rather than aggregated datasets would allow researchers to finely investigate the impact of extreme values on the effects of interest (Reiss et al., 1997).

Moreover, in eye-tracking studies the AoI is commonly referred to as the spatial coordinates of the visual area expected to prompt the effects of interest. Specifically, in familiarization paradigms like that used in the current example, an AoI can be arbitrarily chosen as a function of the spatial area including the main manipulation, i.e., visual stimulus. Moreover, the selection of data points registered within the AoI can also be justified by the fact that artifactual changes in pupil size could occur due to eye movements. That is, the size of the pupil could be larger than the changes induced by the manipulation under investigation. Our results showed that filtering data points falling outside the AoI (step 2) influenced the goodness of fit, the explained variance, and the error associated with the regression estimates of the two models. In particular, among those datasets which included extreme yet plausible values, such a second degree of freedom led to a forking path of mutually exclusive interpretations of the impact of novel audiovisual vs. visual stimuli in increasing resource allocation in 12-month-olds. The second degree of freedom also allows us to check whether audiovisual stimuli continued to prompt their effect even when infants looked near and not necessarily at the visual referent. That is, when there is a mismatch between the visual referent and the fixation. Indeed, audiovisual stimuli frequently appears as objects and actions which are displaced in a different space or time, and infants might use smart strategies to memorize associations between visual objects and the associated audio information, with no need to fixate the visual referent while paying attention to the auditory stimulus (Waxman & Gelman, 2009). Of note, another potentially best practice useful to deal with the noise introduced by gaze shift within the AoI is to include Gaze X and Y coordinates as an additional bivariate smoother term in the GAMMs models. This possibility might add a further forking path to the cognitive pupillometry multiverse that can enrich the knowledge on the robustness of the effect under scrutiny (for a detailed debate, see Van Rij et al., 2019).

Third, the interpolation of blink data has been found to have a differential impact on statistical models that include time as a smoother and those that do not (Hepach and Westermann, 2016; Mathôt et al., 2018; Sirois and Brisson, 2014). While the goodness of models that include time as a smoother remained unaffected, the linear model without smoothers was found to be impacted by the interpolation of blink data. This finding suggests that when blink data is interpolated in the absence of smoothers, it increases the plausibility of statistical estimates. This underscores the importance of accounting for smoothers when dealing with blink data in statistical models. The use of appropriate statistical models can enhance the accuracy and reliability of data analysis and interpretation. It is worth noting that the differential impact of blink data interpolation on statistical models may also be due to the characteristics of GAM models, which are known to handle missing data better than other types of models. The use of GAM models in statistical analysis may thus be a key factor in the observed resilience of models that include time as a smoother to interpolated blink data. Overall, these findings emphasize the importance of checking and choosing appropriate statistical methods and models to optimize the accuracy and validity of data analysis.

Fourth, correcting data points relative to a baseline is a fundamental step in developmental psychophysiology. It is considered a powerful tool to reduce the impact of random pupil-size fluctuations across subjects and trials within subjects. In other words, ignoring baseline correction means comparing pupil sizes between trials neglecting the random effect introduced by the trial sequence and participants. Nevertheless, there is no gold standard defining a rigid length of the baseline period, it varies from study to study depending on the research question and the specific procedure. Some authors prefer long baseline periods (up to 1 s; in e.g., Laeng & Sulutvedt, 2014), which suffer from pupil size fluctuations. Other authors prefer short baseline periods, which on the other hand are susceptible to recording noise (10 ms, Mathôt et al., 2015, 2018). Thus, it is particularly interesting to include the baseline degree of freedom in a pupillometry multiverse (step 4), because a multiverse approach can deal with such a heterogeneity of choices present in the literature. In addition, plausible effects on pupil size should emerge slowly (i.e., > 200-ms manipulation onset). This is a critical aspect in interpreting cognitive pupillometry results, helping disambiguating baseline artifacts from real effects by simply looking at the timing of the effect (Mathot & Vilotijević, 2022, Mathôt et al., 2018; Hepach and Westermann, 2016). In our example, for the sake of results interpretation, we visually inspected pupil dilation relative to the three baseline corrections (vs. no baseline). In particular, in those datasets including extreme yet plausible values, the three levels of the baseline (16, 100, and 200 ms) were associated with a heterogeneous pattern of results. Although with less impact on the results interpretation compared with the previous steps, the baseline correction also influenced the effects in datasets showing only trimmed values.

Lastly, the evaluation of potential influential cases is a crucial aspect in understanding the strength of the effect under discussion in a pupillometry study. In particular, in the fifth degree of freedom of our multiverse, we focused on influential participants who could drive the effect due to a higher proportion of missing data compared to the rest of the group (30%) to check whether the effect is stable at the group level. This consideration is crucial not only in the methodological investigation of a phenomenon of interest but also in the theoretical understanding of it. Evaluating influential participants helps in identifying the key drivers of the effect by checking the robustness of the effect at the subject level. This step enables developmental psychologists to distinguish between random occurrences and systematic patterns in the data, a crucial aspect given that young participants are likely to behave in response to a number of unexpected internal and external stimuli not considered by the experimenter, thus leading to more accurate conclusions. Notably, missing data are the rule rather than the exception in developmental science, yet they are scarcely taken into serious consideration, that is, they are either discarded or interpolated. In fact, it would be informative to consider missing data as a valuable source when non-attendance behavior is involved, which can offer important insights into infants' attentional processes. That is, blindly interpolating missing data may lead to invalid measurements and misinterpretations in developmental science. On the contrary, by considering the behavior of "not looking" as a meaningful aspect of attention, researchers can enrich their understanding of infants' cognitive functioning and provide a more accurate depiction of infants' attention allocation during experiments. Certainly, in order to attribute missing data to a voluntary behavior of 'non-looking' in infants, it would be ideal to longitudinally monitor or at least increase the measurements, for example by scheduling multiple sessions on different days. This way, the responses and missing data would contribute to a precise assessment of the child's attentional response to a specific task. However, data collection in the laboratory is often costly also for families and reduces the possibility of achieving a representative statistical sample. Future research can make good use of remote eye-tracking technologies (Bánki et al., 2022; Tsuji et al., 2022) to complementarily model the distributions of missing data. This would provide a more objective justification for the inclusion or exclusion of participants from data analyses. In general, reasoning on missing data with a multiverse approach would allow for a more nuanced and thorough analysis, ultimately leading to a more robust and reliable understanding of the underlying dynamics of interest. In the present illustrative example, the inclusion vs. exclusion of participants dramatically impact the pattern of results as shown in the specification curve, in Fig. 9, thus increasing the plausibility of interpreting the results with considerable confidence.

## The impact of the two analytical degrees of freedom: Smoothing and random structure

An analytical strategy that ignores the impact of time might misrepresent pupil dilation as a measure of online processing and attention deployment. The common approach of reducing data to averaged values, and the added problem of multiple statistical tests does not allow to take full advantage of the informativeness of pupillometry data (for a debate see Sirois & Brisson, 2014; Mathot & Vilotijević, 2022). For illustrative purposes, we stressed the importance of an approach based on something that is similar to a functional data analysis of cognitive pupillometry (see also Hershman et al., 2022), and we explored the impact of time using a flexible approach based on splines basis, and jointly with a familiarization scheme block we explored the combined effect on pupil size changes across a multiverse of datasets, assessing the robustness of results. That is, we pushed our data processing multiverse inspection towards an analytical multiverse analysis. By jointly looking at the multiverse of results that we generated from a single data collection, it immediately emerged that including smoothing time as a continuous predictor enriched the information about the effect under scrutiny, compared to models which did not include it. Importantly, the specification curve (Fig. 9) permits to represent the entire range of coefficient estimates proposed by the multiverse analysis for assessing if particular combinations of specifications lead to estimates far from the rest of other specifications. In that sense, multiverse analysis helps researchers to address a certain robustness in their statistical analysis avoiding, at the same time, ​​exploitation of data analysis to discover statistically significant patterns (i.e., p-hacking). Our conclusions are reached by comparing each specification as one of the possible plausible forks in the statistical analysis path. Starting from the present illustrative example, we suggest that reasoning on the effects’ timing increased both the plausibility and the informativity of the study. We stress the relevance of investigating the timing of the effects in pupil size changes to make use of cognitive pupillometry as a tool to build reliable models of attention deployment in infancy.

## Conclusions

Variations in pupil diameter provide a useful indirect measure of the time course of visual attention deployment since infancy (Blaser et al., 2014; Brisson et al., 2013; Sirois & Jackson, 2012; Tamasi et al. 2016). However, data processing and data analysis open a window of degrees of freedom that undermines the reliability of the results. The challenges offered by such decisional latitude need to be shared with the scientific community and the uncertainty of results needs to be discussed from a multiverse perspective. That is, we should approach results by bearing in mind that no practice leads to perfectly clean data, yet it is possible and recommended to explore the impact of preprocessing steps in driving the statistical results (Steegen et al., 2016 Dragicevic et al., 2019).

The goal of our multiverse analysis was to answer the question of whether novel audiovisual (vs. visual) stimuli differently impact attention deployment in 12-month-olds. Our results suggest that the audiovisual block reduced pupil dilation as an early effect and increased pupil dilation as a late effect, compared to the visual block. Our illustrative example explored *how* and *when* specific methodological and analytical decisions can affect results. Whereas accounting for the whole multiverse of possible datasets (and modeling) offered by a single data collection might be impractical and sometimes not useful, dealing with at least a plausible portion of the multiverse space is worthwhile and gives back an indication of the robustness of conclusions. Such a philosophical paradigmatic shift of reporting statistical outcomes would also allow the scientific community to discuss how specific practices can prevent/promote the investigation of a given phenomenon (Harder, 2020). That is, the multiverse approach changes the research focus from the ‘best’ conclusion, toward the robustness of the conclusion across multiple degrees of freedom introduced by data processing and analytical choices. The former traditional approach to data analysis and reporting of results might contribute to an overrepresentation of type I errors (Simmons et al., 2011), while jeopardizing the trust in developmental science. Although a few studies in developmental science (e.g., Oakes et al., 2021; Donnelly et al., 2019) have already adopted a multiverse approach to their empirical investigation, with no study, to our knowledge, having applied it to pupillometry, we strongly encourage future empirical contributions to share both raw data and the degrees of freedom in pupil data management because dealing with such uncertainty would give back a robust understanding of functional interpretation of such a powerful psychophysiological measures in developmental research.

In conclusion, collecting ocular metric measures in infancy and early childhood is a true challenge. On the one hand, recruiting families with infants is a slow process that presents practical obstacles to sampling, such as the time availability of families. Moreover, the success rate of data collection is hindered by the characteristics of this population, which, compared to adult individuals, show greater ease of getting bored during the repeated measures in experimental conditions and prefer to visually explore the environment, sometimes not fully respecting the stable posture that is dear to eye-tracking studies. This, coupled with the great intra- and inter-individual variability of infants and children, certainly introduces multiple sources of error, missing data and drop-out that add to those observed in adult studies. Such uncertainty needs to be declared, accepted, and addressed in order to discuss the results of a study with developmental populations, which, although simple, is not trivial, as hopefully has been presented here. The forking path encountered during the preprocessing steps of the familiarization task indicated that evaluating the impact of extreme values, areas of interest, blink distribution, and missing data distribution over time and per participant becomes necessary to obtain even remotely robust information from a data collection with infants, in general. It is important to note that this type of evaluation helps the field to design optimal experimental conditions, in terms of data collection efficiency that reduce the impact of preprocessing degrees of freedom and increase the robustness of the results. Furthermore, studying data noise and missing data in experimental and controlled studies, taking into account each individual participant, allows for a quantitative appreciation of the large individual difference expected in the early years of life. Thus, exploring the multiverse in pupillometry is likely to be a candidate tool to increase our understanding of individual differences in developmental pathways of attention, learning processes, and beyond.

## Data Availability

The data used in this study are openly available in the OSF repository at [https://osf.io/p8nfh/]. Researchers interested in Supplementary materials to replicate the analysis can find detailed instructions on the repository.
